# Supplementary material for: Moderation analysis of exchange rate, tourism and economic growth in Asia
Source: PLoS One. 2022 Dec 30;17(12):e0279937. doi: 10.1371/journal.pone.0279937 (PMC9803286; doi:10.1371/journal.pone.0279937)
Supplement: Attachment — Submitted filename: review.docx [file pone.0279937.s002.docx]

The paper attempts to examine “moderation analysis of exchange rate, tourism and economic growth in Asia”. After reviewing, I find that this paper is interesting. The paper is readable ragarding the case of economic growth in Asian in the background of exchange rate, tourism and their interactive association.

The methodology of this research is acceptable. The authors applied IV GMM as well as quantiles regression as a consideration for checking. For better contribution to the literature, I have some revisions that are good for enhancing the quality of the manuscript.

1. Table 1 should summarize the source of the data that is good for readers to know.
2. Economic growth can be proxied by few indicators, this paper selected GDP per capita, why other proxies are not considered?
3. 𝑹′𝑖𝑡 should be explained (page 9)
4. the instrumental variables-two-step generalised method of moments (IV-GMM) techniques. Why you do not run for one-step, or system or diff GMM. The research needs to discuss with other techniques related to GMM.
5. How do you know the endogenous problems in the analysis. This should be checked before running GMM. And, is there cross-sectional dependence in this sample?
6. There are some minus values of FDI, I already checked and recognized that the authors had deleted these values. Are the steps acceptable? I think that in the case of lots of minus values, it may nagetively affect the quality of the regression.
