# Supplementary material for: Moderation analysis of exchange rate, tourism and economic growth in Asia
Source: PLoS One. 2022 Dec 30;17(12):e0279937. doi: 10.1371/journal.pone.0279937 (PMC9803286; doi:10.1371/journal.pone.0279937)
Supplement: Attachment — Submitted filename: Responses to Reviewers Comments_ExR-Tour-EG, Asia.docx [file pone.0279937.s003.docx]

**PONE-D-22-18174**

**Moderation analysis of exchange rate, tourism and economic growth in Asia**

**Dear Editor,**

We appreciate the opportunity given to us to address the minor comments raised thereby improving the manuscript. We have indicated changes made in the text with blue prints. Please find herein our point-by-point responses to the Reviewers’ comments.

Reviewer's Responses to Questions

Comments to the Author

1. Is the manuscript technically sound, and do the data support the conclusions?

The manuscript must describe a technically sound piece of scientific research with data that supports the conclusions. Experiments must have been conducted rigorously, with appropriate controls, replication, and sample sizes. The conclusions must be drawn appropriately based on the data presented.

Reviewer #1: Yes

**Response:** We are encouraged by this comment. Thank you.

Reviewer #2: Yes

**Response:** We are encouraged by this comment. Thank you.

Reviewer #3: Partly

**Response:** We appreciate your comment and will do our best to all concerns.

2. Has the statistical analysis been performed appropriately and rigorously?

Reviewer #1: Yes

**Response:** We are encouraged by this comment. Thank you.

Reviewer #2: N/A

Reviewer #3: I Don't Know

**Response:** We appreciate your comment and will do our best to all concerns.

3. Have the authors made all data underlying the findings in their manuscript fully available?

The [PLOS Data policy](http://www.plosone.org/static/policies.action#sharing) requires authors to make all data underlying the findings described in their manuscript fully available without restriction, with rare exception (please refer to the Data Availability Statement in the manuscript PDF file). The data should be provided as part of the manuscript or its supporting information, or deposited to a public repository. For example, in addition to summary statistics, the data points behind means, medians and variance measures should be available. If there are restrictions on publicly sharing data - e.g. participant privacy or use of data from a third party - those must be specified.

Reviewer #1: Yes

**Response:** We are encouraged by this comment. Thank you.

Reviewer #2: Yes

**Response:** We are encouraged by this comment. Thank you.

Reviewer #3: No

**Response:** We appreciate your comment and will do our best to all concerns.

4. Is the manuscript presented in an intelligible fashion and written in standard English?

PLOS ONE does not copyedit accepted manuscripts, so the language in submitted articles must be clear, correct, and unambiguous. Any typographical or grammatical errors should be corrected at revision, so please note any specific errors here.

Reviewer #1: No

**Response:** We appreciate your comment and will do our best to all concerns.

Reviewer #2: Yes

**Response:** We are encouraged by this comment. Thank you.

Reviewer #3: Yes

**Response:** We are encouraged by this comment. Thank you.

5. Review Comments to the Author

Please use the space provided to explain your answers to the questions above. You may also include additional comments for the author, including concerns about dual publication, research ethics, or publication ethics. (Please upload your review as an attachment if it exceeds 20,000 characters)

**Reviewer #1:** Idea of the paper and statistical parts have been performed appropriately and rigorously. And there is no problem. But some formal parts have mistakes and errors. Some of them:
**Response:** We are encouraged by your comment. Thank you very much. We will do our best to address your concerns thereby improving the quality of the manuscript.

1- Abstract part should be improved.
**Response:** The Abstract is recast.

2- Too many references. Can be reduced according to the journal index (Scopus, Sci, ssci etc.)
**Response:** We cited relevant literature to support our assertions and choice of variables. We avoided predatory articles as all references are indexed in Scopus, Web of Science, SSCI, ESCI etc.

3- No need to citations in Data and Methodology parts. They must be in Literature Review part.
**Response:** We appreciate this but humbly differ on your position. Citations to support choice of variables or methodologies are in line with industry practice and they lend credence to the variables and techniques used in the study.

4- In text there are citation errors. For example more than 3 authors use et al. Some parts it is true but some parts wrong.
**Response:** We appreciate this and have aligned the in-text citations such that *“et al.”* is used where there are more than 3 authors.

5- Some citations are missed in references especially in page 12.
**Response:** We are very grateful for this observation and have now updated the reference list. The updates are indicated with yellow highlights.

6- Use "literature review" instead of "Review of literature".
**Response:** Corrected to “Literature Review”.

7- At conclusion part comparisons with previous studies can be made.

**Response:** We appreciate this comment and have recast the CONCLUSION as suggested. Kindly see the blue texts.

**Reviewer #2:** The paper attempts to examine “moderation analysis of exchange rate, tourism and economic growth in Asia”. After reviewing, I find that this paper is interesting. The paper is readable regarding the case of economic growth in Asian in the background of exchange rate, tourism and their interactive association.
**Response:** We are encouraged by this feedback and we will do our best to address all concerns raised.

The methodology of this research is acceptable. The authors applied IV GMM as well as quantiles regression as a consideration for checking. For better contribution to the literature, I have some revisions that are good for enhancing the quality of the manuscript.

1. Table 1 should summarize the source of the data that is good for readers to know.

**Response:** We appreciate this comment and have indicated World Bank (2020) as “Source” in the NOTE to Table 1.

2. Economic growth can be proxied by few indicators, this paper selected GDP per capita, why other proxies are not considered?

**Response:** Yes, this is true but being a panel data study comprising different countries with different economy and population size; we align with related studies (see cited references in section 3.1) to use real GDP per capita which theoretically provides the amount of money that each person gets in that country. It helps to capture living standards and it is an indirect measure of per capita income.

3. 𝑹′𝑖𝑡 should be explained (page 9)

**Response:** 𝑹′ is now defined as vector of vector of control variables in natural logarithms. Kindly see the note below Equation [3].

4. the instrumental variables-two-step generalised method of moments (IV-GMM) techniques. Why you do not run for one-step, or system or diff GMM. The research needs to discuss with other techniques related to GMM.

**Response:** We appreciate this comment and humbly state that as explained in Section 3.3, this study deployed the IV-GMM technique which is quite common and relevant among researchers due to its features which includes correcting for cross-sectional dependence, endogeneity, autocorrelation and heteroscedasticity in the data. The IV-GMM variant used is developed by Baum et al (2007a, b). Kindly see cited references in Section 3.3. We further explained that: “*The routine performs several variants of single-equation linear regression models including the generalized method of moments (GMM). Hence, the GMM variant which implements the two-step feasible GMM estimation (that is, gmm2s option) is adopted to ensure that our results are devoid of endogeneity, heteroscedasticity and autocorrelation”.* We restricted to using the IV-GMM technique because it suits our analytical procedure.

5. How do you know the endogenous problems in the analysis. This should be checked before running GMM. And, is there cross-sectional dependence in this sample?

**Response:** Given that all economic variables cannot be included in a regression, there is the likelihood of endogeneity (correlation between explanatory variable and the error term) occurring. Hence, it is advisable to always use estimation techniques that control for this problem. So, even without testing for endogeneity and/or cross-sectional dependence, the deployment of the IV-GMM gives assurance that our estimations are devoid of these problems.

6. There are some minus values of FDI, I already checked and recognized that the authors had deleted these values. Are the steps acceptable? I think that in the case of lots of minus values, it may nagetively affect the quality of the regression.

**Response:** We appreciate this statement but humbly state that your assertion is not true. We did not alter the data used for our analysis. As proof, we uploaded the underlying data and the FDI variable is coded as *fdinibp*. If you look closely, you will observe negative values of the variable as obtained from World Bank (2020) World Development Indicators.

**Reviewer #3:** The paper under consideration looks at the impact of tourism on GDP growth and the interactions of the impact with exchange rate. In my opinion this paper has important shortcomings that will prevent it from being published in the current form. My suggestion is rejection. The issues that lead to my decision are as follows:
1. The paper largely ignores the growth regression literature and certainly aims to be a part of it.
**Response:** We appreciate this comment and hope that given the revisions made to this manuscript, you will reconsider your position and give us the opportunity to publish our work in this esteemed Journal.

**Response:** We humbly address your concern by stating that TOURISM and not GROWTH is the main focus of this study. Though, economic growth is the outcome variable, our emphasis on tourism receipts (main explanatory variable) is deliberate. From the INTRODUCTION to the CONCLUSION, the emphasis on TOURISM is to situate the study objective which is evaluate the effect of tourism on economic growth and if the effect is influenced by exchange rate.

2. The value added generated in the tourism sector is in fact part of the overall value added of the economy. This is largely correlated with the international tourism. What sense does it have to regress GDP on a component of it? We can find out quite precisely what is the EXACT contribution of tourism to GDP and GDP growth.
**Response:** We appreciate this comment and humbly state that every economic variable is somewhat correlated. However, engaging the regression of economic variables does not lead to bias once the level of correlation is below the allowed threshold of 0.80 from where collinearity becomes a problem. Unfortunately, the data on the exact contribution of tourism is not available hence, this study aligned with related studies to use the composite tourism variable. Kindly see cited references in section 3.2.

3. The models are estimated by GMM. However, what are the instruments? The paper does not seems to use any sort of Arellano-Bond, Arellano Bower System-GMM. So the description is vague. And in particular, the panel System-GMM methods are mainly used to solve the endogeneity caused by the lagged dependent variable and not the inherent endogeneity of the economic problem posed here. So this part clearly needs clarification and justification. It does not suffice to write that „results are devoid of endogeneity, heteroscedasticity and autocorrelation.”
**Response:** We thank you for this comment. This study did not use Arellano-Bond, Arellano Bover System-GMM. As explained in Section 3.3, this study deployed the IV-GMM technique which is quite common and relevant among researchers due to its features which includes correcting for cross-sectional dependence, endogeneity, autocorrelation and heteroscedasticity in the data. Kindly see cited references in Section 3.3. About instrumentation, tourism and exchange rate are instrumented with their first difference and level terms. We have now indicated these in Footnote 4.

4. The measurement of both TRPT and GDP in USD should be discussed, i.e., the volume of tourist services may be positively related to depreciating exchange rate but its value in USD may not.
**Response:** We appreciate this comment and make these clarifications.

1) This study used GDP per capita (constant, US$) which is the real per capita income.
2) From World Bank (2022) World Development Indicators, International tourism, receipts (% of total exports) is defined as: **expenditures by inbound visitors, including payments to foreign carriers for international transport**. In other words, this composite variable captures the spendings/expenditures of inbound tourists to Asia, among others. We have now stated this as Footnote 3.
3) Also, from Table 2, the positive correlation coefficient between tourism receipts and the exchange rate is 0.169 which is below the threshold of 0.80 from where collinearity becomes a concern.

5. To what extend this is a different problem than analysis of any export-oriented sector? Why do we care?

**Response:** Though, the tourism variable used in this study captures a segment of the export sector, we explain that our study differs from related export-oriented analysis by evaluating if the exchange rate influences the tourism-growth nexus. To the best of our knowledge, this is the first paper to evaluate this interwoven nexus.

6. How about controlling for real exchange rate, standard in BOP and competitiveness-related studies.

**Response:** We appreciate this comment and humbly state that our study brought novelty to the tourism-growth by examining the influence of exchange rate. Thus, given available data, future studies may control for the real exchange and competitiveness to expand the frontier of knowledge. We have included this in the concluding part of Section 5.
