# Supplementary material for: Moderation analysis of exchange rate, tourism and economic growth in Asia
Source: PLoS One. 2022 Dec 30;17(12):e0279937. doi: 10.1371/journal.pone.0279937 (PMC9803286; doi:10.1371/journal.pone.0279937)
Supplement: Attachment — Submitted filename: 2nd_Responses to Reviewers Comments_ExR-Tour-EG, Asia.docx [file pone.0279937.s004.docx]

**PONE-D-22-18174R1**

**Moderation analysis of exchange rate, tourism and economic growth in Asia**

**Dear Editor,**

We appreciate the second opportunity given to us to address the minor concerns of the 3^rd^ Reviewer. Please find herein our point-by-point responses to the comments. We have indicated these points in the manuscript with blue prints.

**Comments to the Author**

**Reviewer #1:** This study brings novelty to the tourism literature by re-examining the role of exchange rate in the tourism-growth nexus. It differs from previous tourism-led growth narrative to probe whether tourism exerts a positive effect on economic growth when the exchange rate is accounted for. All corrections are enough for publishing.

**Response:** We are encouraged by this comment. Thank you.

**Reviewer #2:** Dear Authors,
I feel satisfied with this version and your replies on my comments. Therefore, I have decided the acceptance for this paper.
Best,

**Response:** We are encouraged by this comment. Thank you.

**Reviewer #3:** I do not believe that the authors have taken my comments seriously. It is not enough to say: "we are using GMM" because it is a large class of models. What are the instruments? What are the moment conditions? Authors say they do not use GMM based on lagged values (Arellano Bond, Arellano Bover) but another approach. So what exactly is it?

**Response:** We appreciate your feedback but:

(1) Humbly state that at no point did we mention *“we are using GMM because it belongs to a class of models”*. This statement cannot be credited to our submissions (initial and revised) as we never mentioned anything of this sort.
(2) Humbly refer you to your initial comment (see below) and our response where we responded by stating that we did not use Arellano-Bond, Arellano Bower type-GMM but IV-GMM.
(3) Mentioned that we used IV-GMM variant developed by Baum et al (2007a, b). Kindly see cited references in Section 3.5. We further explained that: “*The routine performs several variants of single-equation linear regression models including the generalized method of moments (GMM). Hence, the GMM variant which implements the two-step feasible GMM estimation (that is, gmm2s option) is adopted to ensure that our results are devoid of endogeneity, heteroscedasticity and autocorrelation”.* We restricted to using the IV-GMM technique because it suits our analytical procedure.

On instrumentation, we responded to you that tourism and exchange rate are instrumented with their first difference and level terms (as stated in Footnote 4).

**Dear 3^rd^ Reviewer, this was your initial comment on the technique of estimation:**

3. The models are estimated by GMM. However, what are the instruments? The paper does not seems to use any sort of Arellano-Bond, Arellano Bower System-GMM. So the description is vague. And in particular, the panel System-GMM methods are mainly used to solve the endogeneity caused by the lagged dependent variable and not the inherent endogeneity of the economic problem posed here. So this part clearly needs clarification and justification. It does not suffice to write that „results are devoid of endogeneity, heteroscedasticity and autocorrelation.”

**And this was our response to your initial comment:**

We thank you for this comment. This study did not use Arellano-Bond, Arellano Bover System-GMM. As explained in Section 3.3 (initial submission), this study deployed the IV-GMM technique which is quite common and relevant among researchers due to its features which includes correcting for cross-sectional dependence, endogeneity, autocorrelation and heteroscedasticity in the data. Kindly see cited references in Section 3.5 (revised submission). About instrumentation, tourism and exchange rate are instrumented with their first difference and level terms. We have now indicated these in Footnote 4.
