# Supplementary material for: Moderation analysis of exchange rate, tourism and economic growth in Asia
Source: PLoS One. 2022 Dec 30;17(12):e0279937. doi: 10.1371/journal.pone.0279937 (PMC9803286; doi:10.1371/journal.pone.0279937)
Supplement: Attachment — Submitted filename: 3rd_Responses to Reviewers Comments_ExR-Tour-EG, Asia.docx [file pone.0279937.s005.docx]

**PONE-D-22-18174R2**

**Moderation analysis of exchange rate, tourism and economic growth in Asia**

**Dear Editor,**

We appreciate the third opportunity given to us to address the minor concerns of the 3^rd^ Reviewer. Please find herein our point-by-point responses to the comment. We have indicated these points in the manuscript with blue prints.

**Comments to the Author**

**Reviewer #3:** Now with the paper specifying the actual method and the choice of instruments, the only thing that is lacking is providing test statistics to check for the validity of instruments, i.e., the J statistic or equivalent and the discussion on the validity of instruments. The authors seem to treat the IV-GMM technique as a solution to several econometric problems but they do not check whether the problems have been indeed solved.

**Response:** We appreciate your feedback and have now updated Table 3 with the diagnostics from the Hansen-J statistic. We also offered the below explanation in the manuscript (indicated in blue prints):

*“On the reliability of the instruments used to validate the robustness of our estimations, we controlled for identification and exclusion restrictions which are indispensable for robust GMM estimations [12,13]. Having used the IV-GMM estimation in ivreg2, the appropriate test of overidentifying restrictions and testing the validity of instruments used is the Hansen J statistic: the GMM criterion function. From the lower panel of Table 3, the p-value of the Hansen-J statistic across the six models ranges between 0.085 and 0.3874 which is clearly above 0.05. Hence, it fails to reject the null hypothesis of instruments validity indicating that the instruments used are valid and robust to our analysis”.*
